# Transcriptional profiling of olfactory system development identifies *distal antenna* as a regulator of subset of neuronal fates

**DOI:** 10.1038/srep40873

**Published:** 2017-01-19

**Authors:** Scott Barish, Qingyun Li, Jia W. Pan, Charlie Soeder, Corbin Jones, Pelin C. Volkan

**Affiliations:** 1 Duke University, Department of Biology, Durham, NC, USA; 2 University of North Carolina- Chapel Hill, Integrative Program for Biological & Genome Sciences, Chapel Hill, NC, USA; 3 University of North Carolina- Chapel Hill, Department of Biology, Chapel Hill, NC, USA; 4 Duke Institute for Brain Sciences, Durham, NC, USA.

## Abstract

Drosophila uses 50 different olfactory receptor neuron (ORN) classes that are clustered within distinct sensilla subtypes to decipher their chemical environment. Each sensilla subtype houses 1–4 ORN identities that arise through asymmetric divisions of a single sensory organ precursor (SOP). Despite a number of mutational studies investigating the regulation of ORN development, a majority of the transcriptional programs that lead to the different ORN classes in the developing olfactory system are unknown. Here we use transcriptional profiling across the time series of antennal development to identify novel transcriptional programs governing the differentiation of ORNs. We surveyed four critical developmental stages of the olfactory system: 3rd instar larval (prepatterning), 8 hours after puparium formation (APF, SOP selection), 40 hrs APF (neurogenesis), and adult antennae. We focused on the expression profiles of olfactory receptor genes and transcription factors—the two main classes of genes that regulate the sensory identity of ORNs. We identify distinct clusters of genes that have overlapping temporal expression profiles suggesting they have a key role during olfactory system development. We show that the expression of the transcription factor *distal antenna (dan*) is highly similar to other prepatterning factors and is required for the expression of a subset of ORs.

Deciphering complex environmental cues requires a high level of functional diversity of the neurons in both the peripheral and central nervous systems. Little is known however about the developmental processes generating this diversity. Sensory systems, particularly the olfactory system, are prime examples of neuronal diversity and how this diversity is required for the survival and reproduction of an organism. The olfactory system for example, is a critical component of behavioral circuits that underlie such behaviors as: foraging, danger avoidance, courtship and parenting.

Drosophila has 50 olfactory receptor neurons (ORNs) that are housed on two olfactory appendages, the antenna and maxillary palps. These 50 classes of ORNs are organized in clusters of 1–4 ORNs in stereotypical combinations within individual sensory hairs called sensilla that cover the surface of the two olfactory appendages^1^,^2^,^3^. There are approximately 1300 and 130 neurons per antenna and maxillary palp respectively^2^,^4^. Each neuron expresses typically a single receptor from a genomic repertoire of approximately 80 possible receptor genes, which belong to one of two gene families, the odorant receptors (OR) and ionotropic receptors (IR)^1^. The ORNs housed in morphologically distinct basiconic and trichoid sensilla types on the antenna and palps express OR genes, except for two ORN classes (Gr21a/Gr63a- and Or10a/Gr10a-expressing neurons), which express gustatory receptors (GRs)^5^,^6^. Neurons housed in coeliconic sensilla usually express ionotropic receptors (IRs), another family of chemosensory receptors^7^,^8^,^9^.

The antenna, like other adult fly appendages, develops from the larval imaginal disc. The antennal disc is specified by the expression of *homothorax (hth*), *distal-less (dll*), and *extradentical (exd*)^10^,^11^. The antennal disc undergoes three major phases of development: prepatterning, precursor selection, and neurogenesis^12^. The epithelial cells of the antennal disc are initially patterned by gene regulatory networks that establish multiple ring-like fields with different and restricted differentiation potentials^13^,^14^,^15^,^16^. Each field gives rise to the precursors that will generate different combinations of ORNs. Sensory organ precursors (SOPs) are selected by the expression of one of the two proneural genes, *amos* and *atonal (ato)*^17^,^18^,^19^,^20^. Finally, the SOPs undergo asymmetric cell division, mediated by Notch signaling, to generate all of the cells (both the neuronal and supporting cells) that make up each sensillum^21^,^22^. During these final divisions, genes that directly regulate olfactory receptor expression act as terminal selectors to determine the final fate of the ORNs^23^,^24^,^25^,^26^,^27^.

Development of ORN identities in the antennae occurs in a step-wise and context dependent nature. This is an inherently temporal process, in which time defines the context of development. Unfortunately, the step-wise, time and context dependent nature of the developmental process makes identifying and understanding the key players in this processes a challenge. Despite a number of mutational studies looking into the regulation of ORN development^14^,^16^,^17^,^18^,^23^, a large majority of the transcriptional programs that lead to the culmination of 50 different ORN classes in the developing olfactory system are unknown.

Here we specifically generated transcriptional profiles from samples collected at four critical developmental stages of the olfactory system: 3^rd^ instar larval (prepatterning), 8 hours after puparium formation (APF, SOP selection), 40 hrs APF (neurogenesis), and adult antennae. This study is a unique, in-depth analysis of the olfactory tissue transcriptome across development. These data provide a broad and unbiased view into the genes that govern and shape diversification of ORNs. We particularly followed the expression profiles of two main classes of genes that can regulate the sensory identity of ORNs: olfactory receptor genes, and transcription factors. These studies revealed distinct clusters of genes with overlapping temporal expression profiles that we interpret in light of the events set to occur during olfactory system development.

## Results

### Stage specific analysis of developing olfactory system transcriptome

**Figure 1:**
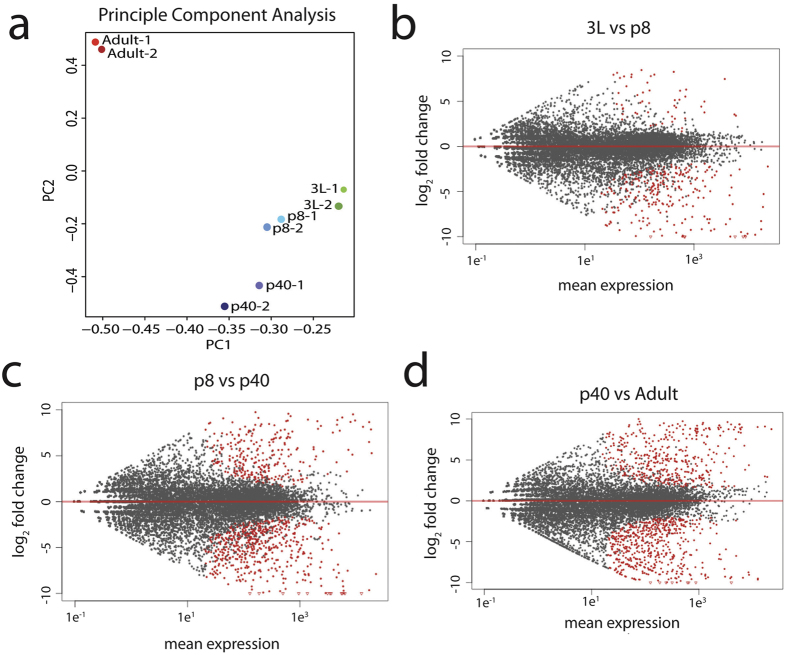
PCA analysis and MA plot comparisons of gene expression across all time points and pairwise comparisons respectively. (**a**) PCA analysis of all four transcriptional time points. The adult transcriptome appears the most divergent among the developmental time points, whereas the 3 L and p8 timepoints show the highest similarity. (**b–d**) MA analysis showing differentially expressed genes between developmental stages (p < 10^−6^), which are highlighted in red.

To analyze the transcriptional dynamics of olfactory system development *in Drosophila melanogaster*, mRNA was sequenced from developing antennal discs and antennae at four different stages (3L, p8, p40, adult). For every time point, we generated two biological replicates, each comprised of RNA pooled from >50 *w1118* flies^13^. We first performed principal component analysis (PCA) to assess the sources of variation of transcripts across RNA datasets from 4 developmental stages (Fig. 1a). Variation detected in the first two principal components (PC1 and PC2) showed high level of similarity between 3 L and p8, both of which significantly differed in global transcription from p40 and adult antennae (Fig. 1a). As expected, the biological replicates for each stage were most similar to each other, segregating away from the transcriptional profiles of other stages (Fig. 1a). The first principal component segregates the samples based on developmental time, such that, the right side of the graph corresponds to earliest developmental stages and the left is the more differentiated states (Fig. 1a). The second principal component clusters the first three time—3L, p8, and p40–points close together while adult antennae are highly segregated by this principle component (Fig. 1a). In other words, each developmental time appeared to have a unique transcriptional profile that clusters away from other stages, especially the adult stage. Given their temporal proximity, it is striking that the p40 and adult antennal transcriptional profiles appeared most distant from each other according to the second principal component (Fig. 1a). We next analyzed which transcripts contribute to PC1 and PC2. Of the >29,000 transcripts that were analyzed only 17 had a correlation coefficient >0.1 for PC1 (Table S1). Three of these transcripts were annotated as having metabolic functions, three as odorant binding proteins (Obps), one as involved in lateral inhibition and 10 had no known function. 21 transcripts had correlation coefficients >0.1 for PC2 (Table S2), and of these 10 were involved in cuticle/chitin development, 8 had no known function and one each were involved in wing disc morphogenesis, lateral inhibition, and metabolism. These results are consistent with the observation that PC2 segregates adult antennae from the other three developmental stages and suggests that the primary transcriptional changes in the antenna that drive late stages of development control the development of the cuticle and adult structures. Given that almost half of the genes identified have no known function suggests that many of the key players in this process remain uncharacterized.

We next analyzed which genes were differentially expressed between each time point, which are represented in MA plots (Fig. 1b–d). Genes that show significant differential expression in each comparison are represented by red dot (p < 10^−6^, Tables S3–S5). This analysis confirmed results from PC analysis: 3L and p8 appeared more similar and segregate from p40 and adult (Fig. 1b–d and Tables S3–S5). These results underscore that the transcriptional profile of the developing olfactory tissue is dynamic across developmental time and that where each stage has a characteristic transcriptional profile. Yet the antenna is a highly complex tissue, and defining the specific cell types undergoing these transcriptional changes requires detailed transcriptome analysis of sorted cell populations.

Analysis of both PCA clustering and MA plots comparing 3L-p8, p8-p40, and p40-adult sequences showed that transcriptional changes occurring in later stage transitions are much more dramatic as compared to earlier stages (Fig. 1a–d). To identify the genes driving transcriptional differences between each time point, we determined enriched functional gene groups among the genes that vary across time points using Gene Ontology (GO) plot package in R (Figs S1–S3). For each comparison we preformed two analyses: using the 500 genes either with the largest fold change or with the lowest p-values (Figs S1–S3). The genes with the largest fold change between 3 L and p8 are enriched for functions in chitin and cuticle development, and the extracellular matrix (Fig. S1A–B and Table S6). Similar enrichment for structural components of chitin, cuticle development, and the extracellular matrix are also detected for the 500 genes with lowest p-values, in addition to genes involved in developmental processes, transcription factor activity and neuronal fate commitment (Fig. S1C,D and Table S6). Between p8 and p40 genes were significantly enriched for changes in the development of the cuticle and chitin synthesis (Fig. S2A,B and Table S7). We also observed that there was enrichment among the genes with the lowest p-values, for cell cycle processes (Fig. S2C,D and Table S7), which is consistent previous observations that all neurons of the antenna are generated by 40hrs APF^15^. Finally, between p40 and Adult transcriptomes differentially expressed genes were enriched for odorant binding and sensory perception (Fig. S3 and Table S8) consistent with the upregulation of OR genes. Metabolic processes were also enriched for genes with the largest fold change between these time points (Fig. S3A,B and Table S8). Interestingly, we also detected enrichment in both groups of genes for plasma membrane structure and function. We observe that processes such as cell cycle regulation, transcription factor activity, and neuronal fate commitment are enriched in the lowest p-value groups but not the largest fold change. This suggests that the largest transcriptional changes occur for genes regulating the development of antennal structure, while other developmental processes are make smaller though significant changes. Overall, these results suggest that earlier stages of antennal development are dedicated for cellular and structural development of the antennal tissue, and the later stages, especially adult stages, are enriched in genes responsible for functional and sensory properties of the antenna.

### Temporal Dynamics of OR Expression

**Figure 2:**
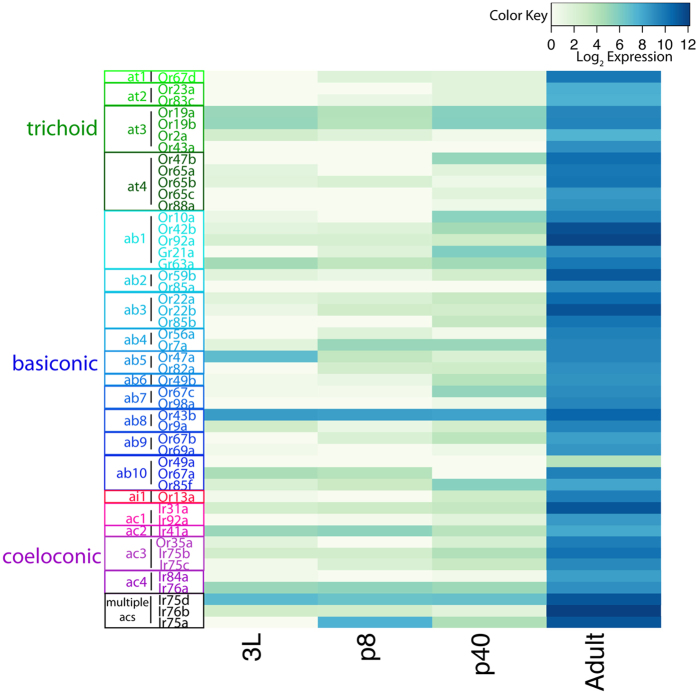
OR/IR developmental expression profiles grouped based on sensilla pairings of ORNs. Expression is graphed with normalized log_2_ expression values, where highly expressed genes are represented in dark blue and low expressed genes are represented with green or white.

Because it has been previously shown that the onset of olfactory receptor expression begins around 40 hrs APF^28^, we were curious to see which, if any, ORs are expressed at this stage. We were able to detect OR transcripts at this stage, but they were expressed at much lower levels as compared to the adult stage (~2 orders of magnitude lower, Fig. 2). Because the expression of ORs at p40 was so low, we wanted to determine if the expression in our RNA-seq data was biologically relevant. We first compared OR expression levels at p40 to the expression of gustatory receptors (GRs). Gustatory receptors primarily function in taste sensation and are expressed predominantly in the labial palps and legs^29^,^30^,^31^. With the exception of the four olfactory GRs (Gr21a, Gr63a, Gr28b, and Gr10a)^32^, GRs are not known to be expressed in the olfactory system, and should therefore not be expressed above background levels in the antenna^33^. The structure of GRs and their expression in gustatory neurons, is relatively similar to ORs in the olfactory organs, except that multiple GRs can be expressed in the same neuron. Most reporters of GR expression show exclusion from the antennae^29^,^30^,^31^,^34^. Using a negative binomial model (Methods), we calculated confidence intervals for each of the 62 antennal ORs to determine if they were above background expression levels. Nearly half (21; 17 ORs and 4 IRs) of the ORs in the olfactory system were expressed at p40 (Fig. 2 and Tables S9 and S10, p > 0.05). Typically, one receptor per senilla subtype was expressed at p40 (Fig. 2 and Tables S9 and S10) with a few exceptions. For instance, ab1 expresses two receptors at p40 and is the only sensilla subtype to house four ORNs, as well as express GRs (Fig. 2 and Tables S9 and S10). In contrast, ab5 and ab8 sensilla also expressed two receptors at p40 and only house two ORN classes. Why specific subsets of the ORs are expressed early in each sensillum remains unknown, but may represent the sequence of developmental and cell-cell communication events as ORNs in the same sensillum pick specific olfactory receptors from limited possibilities.

**Figure 3:**
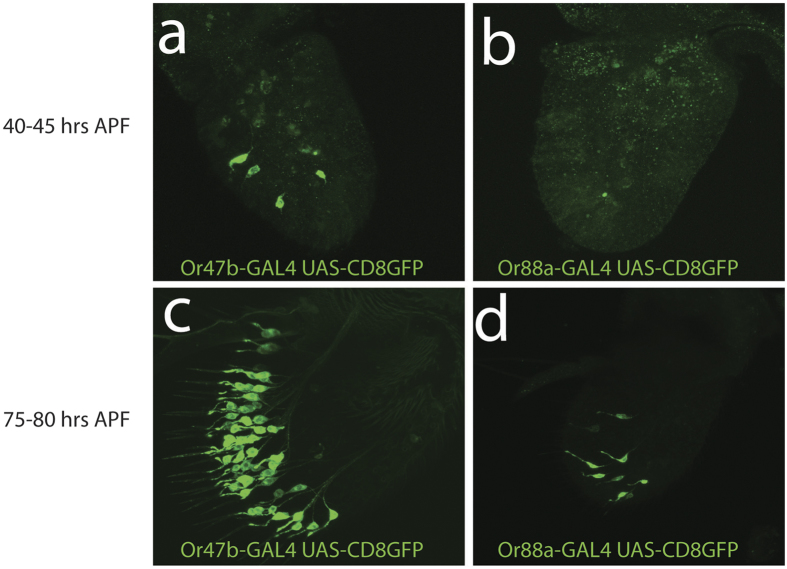
Expression of OR reporters in developing pupal antennae. A small number of neurons express OR47b (top left) at 40–45 hrs APF, whereas OR88a expression in not detected at this stage (top right). By 75–80 hrs APF Or47b expression appears fully developed (bottom left), while OR88a expression is present in only a few neurons (bottom right).

We next wanted to confirm that we could detect expression of ORs at p40 with expression of OR reporters. Well-established OR-GAL4 drivers have been shown to be faithful reporters of OR transcription^1^. Therefore, detecting expression of OR reporters at p40 should provide confirmation of the transcriptome results. For example, in our transcriptome data, among the at4 sensilla OR genes, Or47b is the first to be expressed, followed by the expression of Or65a and Or88a, which are not detected until the adult stage (Fig. 2 and Tables S9 and S10). We confirmed the temporal dynamics of ORs 47b and 88a using OR reporters in the antenna (Fig. 3). The Or47b reporter is expressed at 40 hrs APF, but only in a few neurons (Fig. 3a,c), corresponding to the low transcript levels of Or47b at this stage (Fig. 2). Our results are also consistent with previous reports that show that OR transcript expression level correlates with the number of neurons expressing a given receptor^13^. The early OR expression is also unlikely to be a by-product of the larval olfactory system as the larval olfactory system is not housed in the antennal disc^1^,^35^,^36^ and none of the ORs expressed early are OR genes previously shown to be expressed in the larval olfactory system^1^,^35^,^36^. Or88a expression however is not detected until ~80 hrs APF, by which point Or47b expression has reached the adult level (Fig. 3b,d). ORN fates within each sensilla subtype are defined by whether or not they require Notch for their development (Notch ON or Notch OFF) as they are generated from asymmetric divisions of sensory organ precursors^21^,^22^. We therefore wanted to know whether the ORs that are expressed early shared a common Notch state (i.e. Notch-On of Notch-Off)^21^, which might suggest that they are regulated by the same set of Notch dependent transcription factors. We did not observe any correlation however, among the Notch states of the olfactory receptors expressed at p40, based on previous genetic descriptions (Table S10). In fact, 9 of the ORs expressed at p40 are Notch-On fates and 8 are Notch-Off. So, while there is a slight bias for Notch-On fates, there does not appear to be a clear correlation between Notch state and expression of an OR gene at p40. Based on these criteria, our data also showed that a subset of ORs are expressed at uniformly low levels across the first three stages of development and then sharply increased in the adult. We found that Or19a/b (co-expressed in the same ORN) and Or43b, housed in at3 and ab8 sensilla respectively were expressed throughout development, although at much lower levels than in the adult (Fig. 2 and Table S9).

Our results suggest that there is a temporal sequence to the onset of OR gene expression within a given sensillum, with some OR genes activated early, and others later. We speculate that the temporal sequence of OR expression among the ORNs in the same sensillum might arise as a result of inter-neuron communication that distributes limited OR gene possibilities individually to each neuron. It is possible that the sequence of OR expression is also tied the molecular age of the receptor^37^, with phylogenetically older receptors turning on first and newer ones later. This appears to hold true for both ab1 and at4 sensilla. For ab1 sensilla, Or92a is older than Or10a and is expressed at p40 (Fig. 2 and Tables S9 and S10), whereas we do not detect expression of Or10a until the adult stage (Fig. 2 and Tables S9 and S10). Or42b is older than either of these receptors^37^ and is the first receptor to be expressed in ab1 sensilla (Fig. 2 and Tables S9 and S10). In at4 sensilla, Or47b is older than Or88a and turns on before Or88a^37^ (Figs 2 and 3). Thus, given the dynamic nature of olfactory receptor sequence evolution, the temporal order of olfactory receptor expression within a sensillum might represent the processes that correlate with the evolutionary emergence of new receptors and their integration to the circuits as a new class of ORN.

### Temporal Dynamics of IR Expression

Four of the IRs were expressed at p40: Ir75a, Ir75b, Ir75c and Or35a (An OR expressed in coeloconic sensilla) (Fig. 2). Ir75a is expressed in multiple sensilla and we, therefore, cannot determine which of the sensilla are expressing this receptor at p40. No Irs were expressed at all stages, but Ir75a was expressed at every stage except for 3L (Fig. 2 and Tables S9 and S10). As was the case for Or19a/b and Or43b, the expression of Ir75a was much lower at early developmental stages and sharply increased at the adult stage (Fig. 2 and Table S9). Each coeloconic sensilla subtype contains at least on receptor that is not expressed in the other sensilla subtype, whereas the others are shared across subtypes^9^,^38^. Interestingly, we only observed expression of ac3 IRs (Or35a and Ir75b/c) at p40 (Fig. 2 and Tables S9 and S10). This suggests that the specification of ac3 sensilla occurs first, whereas other subtypes are specified at a later time than that of OR expressing sensilla.

### Transcription Factor Expression

While it is well understood that transcription factors play a key role in specification of ORN identity at all stages of antennal development^13^,^14^,^17^,^18^,^23^,^24^, the precise timing of the expression of these genes across development is not known. Given the combinatorial nature of ORN identity, it is plausible that combinations of transcription factors accumulate on promoters of genes regulating trajectory of a given ORN class in an additive manner, where newly added transcription factors build on, or complex with, existing transcription factors occupying and maintaining the memory of cellular decisions. It is likely that the current set of transcription factors that regulate ORN specification is incomplete. Thus far, most studies have focused on the regulation of ORs in the basiconic and trichoid sensilla^23^,^26^,^27^ and only a few have examined the regulation of ORs/IRs in coeloconic sensilla^13^,^14^,^18^. Furthermore, these studies have primarily been conducted with biased approaches, focusing only a few ORs at a time. Adopting unbiased approaches for determining which transcription factors may play a role in ORN specification is critical to advancing our understanding of the mechanisms of olfactory system development.

**Figure 4:**
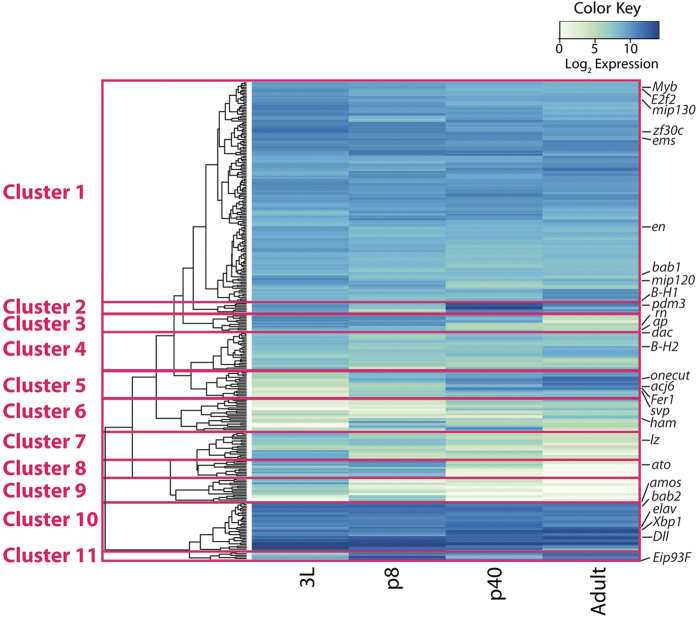
Clustering analysis of ~400 transcription factors based on expression profiles. 11 Clusters were identified based on similarity of expression profiles. Expression is graphed with normalized log_2_ expression values, where highly expressed genes are represented in dark blue and low expressed genes are represented with green or white.

To identify transcription factors expressed during olfactory system development, we queried approximately 400 annotated transcription factors from Flybase and analyzed their expression profiles in our data set (Fig. 4 and Table S11). We observed three patterns of transcription factor expression: those that are expressed early, those that are expressed late, and ones that are expressed throughout all stages (Fig. 4 and Table S11). These expression patterns are also generally predictive of the function of known transcription factors (Fig. S4). Prepatterning factors and proneural genes, such *as rotund (rn*), *apterous (ap), lozenge (lz), amos,* and *atonal (ato)*, are expressed primarily at 3 L and p8 but absent at the p40 and adult stages, consistent with previous reports^14^,^17^,^18^,^39^ (Fig. S4). Whereas terminal selector transcription factors, such as *acj6, fer1, onecut,* and *eip93F*, are highly expressed in p40 and adult antennae but largely absent from 3 L and p8 antennal discs (Fig. S4), consistent with their function as direct regulators of OR expression^23^. Other transcription factors from both the prepattering network and the terminal selectors (*Distal-less (Dll), Bar-H1/2 (B-H1/2), dachshund (dac), bric-a-brac1/2 (bab1/2), xbp1* and *zf30c)* however, are expressed throughout development (Fig. S2 and Table S8)^15^. It is unclear what function these genes have outside of their already well-defined roles in the development of the antennal disc^11^,^13^,^23^,^40^.

#### Clustering Analysis

Because we observed a correlation among known transcription factors between their function and expression pattern, we reasoned that global analysis of the expression patterns of transcription factors could yield novel candidate genes that govern ORN identity. Hierarchical clustering analysis based on developmental expression profile uncovered groups of genes that likely have similar functions (Fig. 4). Transcription factors were clustered based upon their average expression level and pattern across all stages. Clustering analysis revealed 11 clusters of transcription factor expression in the olfactory system (Fig. 4 and Table S12). The majority of known regulators of olfactory system development do not cluster together. Instead they segregate into separate groups, revealing more refined patterns of expression that may be predictive of function. Several clusters stood out because of their pattern and the genes that cluster within them. Cluster 3 contains genes that are primarily expressed early and their expression decreases or is absent at p40 and in the adult (Fig. 4). This cluster contains *rn* and *ap* (Fig. 4 and Table S12), both of which are critical prepatterning factors that regulate ORN identity^13^,^14^. This cluster also contains genes known to play a role in imaginal disc and antennal development, such as *twin of eyeless (toy), teashirt (tsh), distal antenna (dan),* and *distal antenna related (danr)* (Fig. 4 and Table S12)^41^,^42^. It is possible that genes in this cluster play a role in prepatterning of the antennal disc and control ORN specification.

Cluster 5 contains three terminal selector genes *onecut, 48 related 1 (Fer1),* and *abnormal chemosensory jump 6 (acj6),* as well as *seven up (svp), prospero (pros),* and the glial marker *reversed polarity (repo)* (Fig. 4 and Table S12), all of which function primarily in later stages of olfactory system development^23^,^43^. These genes are all lowly expressed in larval antennal discs and their expression increases over time and are highly expressed in adult antennae (Fig. 4 and Table S11). Interestingly, *POU-domain protein 2 (pdm2)* is present in this cluster, which may suggest that it functions as a terminal selector, particularly because other POU-domain transcription factors, *acj6* and *pdm3,* are known to regulate OR expression and connectivity (Fig. 4 and Table S12)^24^,^27^.

Thus far, two major proneural genes, *amos* and *atonal*, have been identified to regulate development of basiconic/trichoid and coeloconic sensilla, respectively^17^,^18^,^19^. In agreement with their role in precursor selection, we found that both *ato* and *amos* expression peaks at p8 in our data (Fig. 4 and Table S11). Genes in cluster 8 are expressed almost exclusively at 3 L and p8 (Fig. 4). Most of the genes in this cluster are also known to function as proneural genes and in Notch-Delta signaling, such as *acheate (ac), scute (sc), asense (ase), senseless (sens), atonal (ato),* and *cousin of atonal (cato)* as well as some enhancers of split (*E(spl))* (Fig. 4 and Table S12)^44^,^45^,^46^,^47^. Of these genes, only *sens* and *ato* have been shown to function in the olfactory system^48^. Investigating the effects of these genes on ORN and sensilla development will be critical in the future.

Interestingly, four *E(spl)s* were present in Cluster 8: *E(spl)-m6, m8, mdelta,* and *mgamma* (Fig. 4 and Table S12). Three other *E(spl)* genes, *E(spl)-m7, m3* and *mbeta,* were detected in our data set but did not cluster in Cluster 8 (Fig. 4 and Table S12). *E(spl)m3* and *mbeta* were present in Cluster 1 and were expressed at all developmental stages (Fig. 4 and Table S11). *E(spl)m7* was present in Cluster 3 and was expressed at 3 L, p8, and p40, but it’s expression decreased in the adult. At early pupal stages, Notch mediates the selection sensory organ precursors and the expression of proneural genes. Whereas, during neurogenesis, Notch signaling segregates binary cell fates during asymmetric divisions of sensory organ precursors to generate each sensillum^17^,^21^,^22^. *Notch, delta* and *serrate* are all expressed throughout development in our analysis (Table S13). It is plausible to imagine that the expression of different *E(spl)* genes or different combinations underlie the different and context-dependent roles that Notch singaling plays during olfactory system development.

### Confirmation of Expression of Bar, Ap, Bab, and Dan

**Figure 5:**
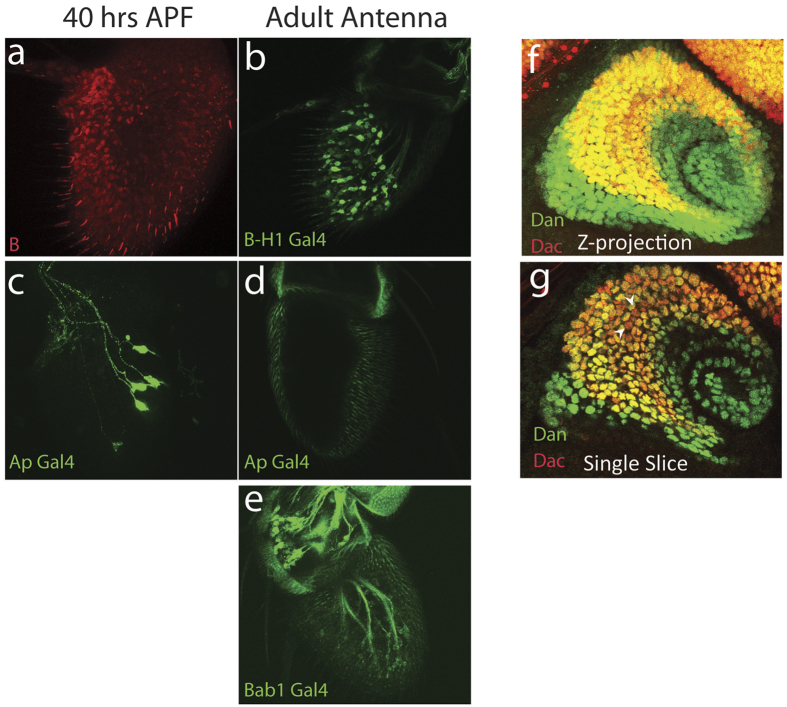
Confirmation of RNA-seq with immunofluorescence and reporter expression. (**a–c**) Expression of BarH-1 (**a**), Ap (**b**), and Bab1 (**c**) in p40 antennae (left) and adult antennae (right). (**d,e**) Expression of *Distal antenna* (Dan, green) in the antennal disc. Immunostaining for Dan show that it is broadly expressed throughout the antennal disc as has been previously reported. Single slices (**e**) also show that Dan is not expressed at the same level in all cells. White arrowheads highlight cells that show low Dan expression.

We next confirmed expression of several transcription factors in the olfactory system using immunofluorescence and imaging of reporters. We have previously published that the transcription factors *BarH1/2 (B-H1/2), apterous (ap),* and *bric-a-brac1/2 (bab1/2)* are expressed in the antennal disc and are critical regulators of ORN identity^13^. In the RNA-seq data, we detected expression of *B-H1/2* and *bab1/2* at p40 and the adult (Fig. 4 and Table S11). *Ap* expression however, is mostly absent at both of these stages in antennal transcriptomes (Fig. 4 and Table S11). Immunostaining for *B-H1/2* was detected broadly in the antenna and expression of the *Bar-H1-Gal4* was present in adult antennae, consistent with the transcript data (Fig. 5a,b). Expression of *ap-Gal4* was present in a small number of ORNs at p40, but was entirely absent in adult antennae, as observed in the RNA-seq data (Fig. 5c,d). We also detected weak expression of the *bab1-gal4* in adult antennae, verifying our transcriptomic analysis (Fig. 5e).

As mentioned above, we detect expression of *dan* mRNA at 3L and p8 in our RNA-seq data (Fig. 4 and Table S11) and we were able to confirm this and previous reports of expression with immunofluorescence in the antennal disc (Fig. 5f). Dan is expressed throughout the entire portion of the antennal disc that specifies the third segment of the antenna (Fig. 5f). We also observe that *dan* expression is not uniform in all cells across the disc, and that some cells express high levels of dan and some barely have any fluorescent signal (Fig. 5g).

### Dan Functions to Regulate the Expression of a Subset of Olfactory Receptors

**Figure 6:**
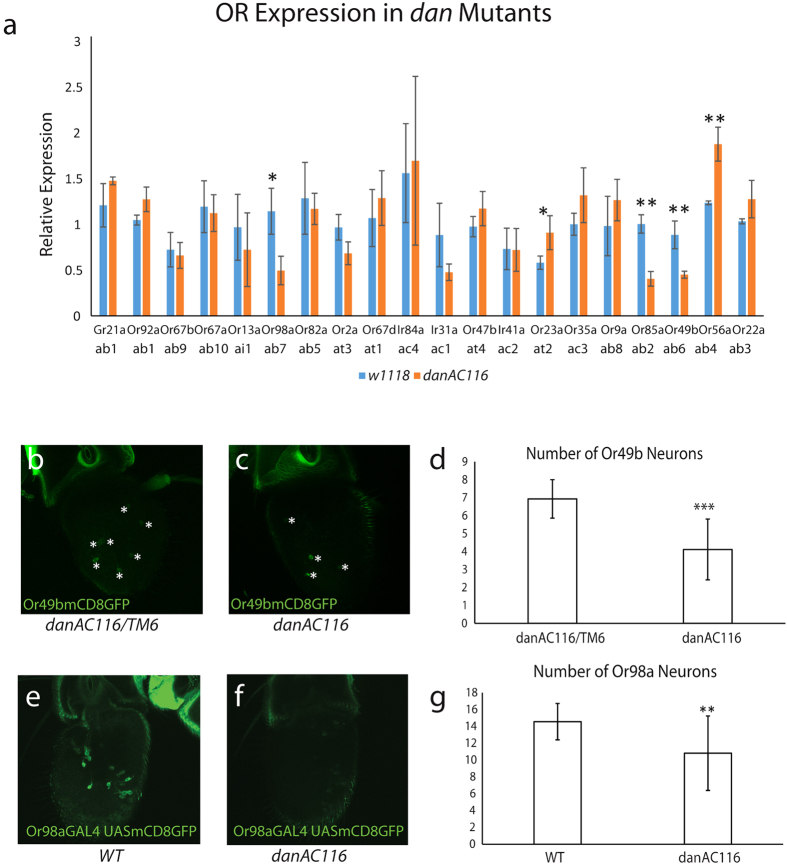
Dan is required for the expression of a subset of ORs. (**a**) qRT-PCR of OR genes as a readout of ORN populations in antennal samples from wildtype and *dan*^*AC116*^mutant flies. Five receptors (Or98a, Or23a, Or49b, Or56a, and Or85a) showed a statistically significant misregulation in *dan* mutants. (**b–g**) Confirmation of qPCR results for Or49b and Or98a by antennal reporter imaging. An average of seven Or49b neurons were present in heterozygous antennae (**b,d**) and an average of four were present in mutant antennae (**c,d**). An average of 15 Or98a neurons were present in wildtype antennae (**e,g**) and an average of 10 neurons were present in *dan* mutant antennae (**f,g**). *p < 0.05 **p < 0.01 ***p < 0.001.

It has been previously reported that loss of *dan* function leads to the production of ectopic hairs on the 3^rd^ segment of the antenna^42^,^49^. In addition, overexpression of *dan* in the leg disc causes the claw to develop into an arista like structure^42^. Because *dan* was present in Cluster 3 and is expressed in a highly similar pattern to prepatterning factors like *rn* and *ap* (Fig. 4), we were curious as to whether Dan may also regulate the development of ORNs as well as larger structures of the antenna. Using qPCR, as published previously by our lab, we measured the expression of 20 OR genes representing at least one receptor that is expressed in each of the sensilla subtypes on the antenna in wildtype and *dan* mutant flies. We detected four ORs that were significantly down-regulated and three that were significantly up-regulated (Fig. 6a). There does not appear to be any particular pattern of those ORs.

We were able to confirm our qPCR results by imaging of OR reporters in the antenna (Fig. 6b–g). We observed a statistically significant decrease in the number of Or49b and Or98a ORNs in *dan* mutants (Fig. 6b–g). It is plausible to think that different sensilla subtypes are specified by the expression of both *dan* and *danr*, but some subtypes are more sensitive to changes in *dan* expression. Correspondingly, we see some cells that express Dan at lower levels than other cells of the antennal disc (Fig. 5g, white arrowheads). It would also be reasonable to think that a distinct subset of ORs would be misregulated in *danr* mutants. It has been previously published that down regulation of *dan* and *danr* via mutation of *ss* leads to an up-regulation of *Antennapedia (Antp)*^42^, which could suggest that in *dan* mutants the antenna has undergone a partial conversion to a leg phenotype and therefore altered the expression of some OR genes.

## Discussion

Here we report a detailed transcriptome analysis of the adult Drosophila olfactory system during development. Our analyses have revealed the temporal dynamics of all antennal olfactory receptor gene expression during ORN development. We also identified transcription factor programs in the developing olfactory system with stage specific functions corresponding to different processes in ORN development. And finally, we show that one of these transcription factors, *distal antenna*, is required for the development of a subset of ORN classes. Although this study is the first to analyze the transcriptome of the olfactory system across developmental stages, the antenna is a highly complex tissue and further cell type specific analyses will likely yield further insights into these processes.

ORNs in the same sensillum arise through asymmetric divisions of a single multipotent precursor cell, yet the timing for terminal differentiation of ORNs, as assayed by the onset of olfactory receptor expression, has not been identified. Our analysis showed that within each sensillum, there is a temporal order to the onset of expression among the olfactory receptor possibilities. That is, onset of olfactory expression occurs earliest in only one of the neurons, followed by other olfactory receptors expressed sequentially, suggesting cell to cell communication among ORNs that determines the temporal order of olfactory receptor expression. Indeed, perturbations in Notch signaling were shown to contribute to the selection of alternate olfactory receptor expression by alternate ORNs in the same sensillum^21^. Upon selection of a “default” olfactory receptor within each sensillum, Notch signaling can relay this information to neighboring ORNs to suppress expression of the “default” receptor and select another olfactory receptor transcriptionally available in the lineage. These modifications might require chromatin regulation, as mutations in *alhambra (alh),* a chromatin modulator, result in acquisition of the default olfactory receptor expression at the expense of alternate olfactory receptors without modifying the target site of ORN axons in the antennal lobe^28^. There might be different spatial and temporal requirements of transcription factor and chromatin complexes for each sensilla lineage, as effects of *alh* mutants are restricted to only few sensilla^28^. The expression patterns of both chromatin modulators and transcription factors in specific cell types remains an understudied area of olfactory system development.

The transcriptional profile of olfactory system development showed dynamic and complex trends in the expression of transcription factors that might function in specification of ORN fates. Three different trends for transcription factor dynamics were detected. Among these, a majority of prepatterning and proneural genes are expressed early and are turned off in later stages of ORN development. Transcripts for a small number of these transcription factors did persist till later stages. Transcription factors previously shown to directly regulate olfactory receptor expression in genetic studies, on the other hand, generally showed a trend towards expression in late developmental stages, with only a few exceptions. Analysis of approximately 400 proteins with transcriptional regulatory roles, revealed other factors that show similar temporal profiles to known factors. One of these factors is *distal antenna (dan),* which has been previously shown to be expressed in many developing neural tissues, including the antennal disc. In addition, it has been to regulate fate specification of neuronal pools from embryonic neuroblasts.

The majority of ORN classes were not affected by loss of *dan*, except for a few classes namely: Or49a, Or56a, Or98a and Or23a, which all showed changes in transcript levels in qRT-PCR from *dan* mutant antennae. Among these, number of Or49b and Or98a ORNs were confirmed to be decreased by antennal reporter imaging, which underlies the decrease in their transcripts in *dan* mutant antennae. Most of the ORs that are affected by *dan* mutation developmentally arise from the center of the antennal disc except for Or98a^13^. Other than this trend, there does not seem to be any clear pattern to which ORs are affected by loss of *dan*. Because of its broad expression pattern, we would have expected a wider array of ORs to have been affected and so it remains unclear why these particular ORs are more sensitive to changes in *dan* expression. It is plausible to imagine that expression of Dan together with the rest of the transcription factor networks patterning the antennal disc in a combinatorial code to determine different precursor fields within the antennal disc. Broad *Dan* expression in the antennal disc is similar to the expression of *bab1* and *bab2,* which also belong to the combinatorial code defining different zones on the antennal disc. Mutations in *bab1* and/or *bab2* also weakly affect only specific ORN fates^13^. Of the ORs that were significantly down-regulated in *dan* mutants, none completely lost expression as has been reported in mutation of other genes that function in prepattering of the antennal disc^13^,^14^. This is likely due to the possible redundant functions of *dan* and *danr* in the antennae^42^,^49^. We would therefore predict to see more dramatic phenotypes and many more ORs affected in *dan-danr* double mutants. Previous work has shown that *dan* and *danr* are critical regulators of antennal identity^42^,^49^. It is possible that they provide a link between the regulation of broad antennal development and the development of specific ORN identities. Understanding the interaction between *dan, danr* and the early prepatterning gene regulatory networks will provide key insights into the specification of different sensilla identities and ORN classes.

## Materials and Methods

### Fly Genetics

*w1118* flies were used for RNA-seq analysis. OR CD8-GFPs and OR-GAL4s were gifts from Leslie Vosshall and Barry Dickson respectively. bab1^Pgal4–2^ (#6803), dan^AC116^ UAS-CD8 GFP and ap^md544^ were acquired from Bloomington Stock Center. NP4099 (Bar^GAL4^) was from Drosophila Genetic Resource Center.

Fly Genotypes

Figure 3a,c. Or47b-Gal4 UAS-CD8GFP

Figure 3b,d. Or88a-Gal4 UAS-CD8GFP

Figure 5a. w^1118^, NP4099 (Bar^GAL4^)/+; UAS-CD8GFP/+

Figure 5b. ap^md544 Gal4^/+; UAS-CD8GFP/+

Figure 5c. UAS-CD8GFP/+; bab1^Pgal4–2^/+

Figure 5d. w^1118^

Figure 6a. w^1118^, dan^AC116^

Figure 6b. Or49bmCD8GFP/+; dan^AC116^/TM6B

Figure 6c. Or49bmCD8GFP/+; dan^AC116^

Figure 6d. Or49bmCD8GFP/+; dan^AC116^/TM6B, Or49bmCD8GFP/+; dan^AC116^

Figure 6e. Or98a-Gal4 UASCD8GFP/+; dan^AC116^/TM6B

Figure 6f. Or98a-Gal4 UASCD8GFP/+; dan^AC116^

Figure 6g. Or98a-Gal4 UASCD8GFP/+; dan^AC116^/TM6B, Or98a-Gal4 UASCD8GFP/+; dan^AC116^

### Immunohistochemistry

Samples were fixed with 4% paraformaldehyde, washed with phosphate buffer with 0.2% Triton X-100, and staining as previously described. rabbit α-Bar-H1 (Tetsuya Kojima) or α-Dan (Minoree Kohwi) was used in a 1:100 and 1:500 concentration, respectively.

### RNA-seq

RNAseq was performed as described before^13^. Wandering third instar larval antennal discs (~70 for each genotype), 8 hr APF pupal antennae (~50 for each genotype), 40 hr APF pupal antennae (~50 for each genotype), and adult antennae (150 males and 150 females) from w^1118^ flies were dissected. We extracted RNA only from the antennal portion of the larval eye-antennal discs in order to remove contamination by transcripts from the developing eye. RNA sequencing libraries were prepared with TruSeq Stranded mRNA Sample Prep Kit (Illumina) following the manufacturer’s instructions. For the RNA fragmentation step, 94 °C, 2 min was used with the intention to obtain a median size ~185 bp. PCR amplification was done with 15 cycles. A total of 24 multiplexed libraries (barcoded) were accessed for quality and mixed altogether before separating to two identical pooled libraries, which are subject to cluster generation followed by Illumina 50 bp paired-end sequencing by UNC High-Throughput Sequencing Facility (HTSF), as described in Li and Barish *et al*.^13^.

### Analysis of RNAseq data

Following Li *et al*.^13^. The *Drosophila melanogaster* transcriptome (r5.57) was downloaded from flybase and bwa indexed was created with bwa-0.7.8. Each sequencing file was aligned to the transcriptome, and. sam files for each sample were generated by putting two alignments from both reads together. At least 80% of the total reads were able to align to the reference sequence. After that, count tables were made for each sample with a customized python script, and further consolidated into a matrix containing transcript ID and read counts from all genotypes for each stage with a Ruby script. These matrices were used as inputs for differential expression analysis using customized DESeq2 R script.

### Estimating the Probability That a Gene Is Expressed

With *in situs* and similar tools it is straightforward to determine if a particular Or is expressed in adult. In the larval stages we need to use the RNAseq data to determine which genes are on or off. Negative binomial distributions are commonly used to model gene expression in RNAseq data. For each stage, we parameterized a negative binomial using the raw count data from non-antennal Gr. These are expected to not be expressed and thus any “counts” associated with these genes should correspond to experimental noise. We then compared the observed levels of Or expression to this distribution. *P*-values corresponding to the chance that the observed level of expression could have occurred by chance under this model were then calculated.

### PCA Analysis

PCA was preformed using the normalized count tables described above and the princ comp function in R.

### Gene ontology analysis

The data files of differential gene expression were processed & filtered with command line tools. Gene Ontology enRIchment anaLysis and visuaLizAtion (GOrilla) analysis^50^ on differentially expressed genes (Fig. S1). We entered the entire 34,946 reference sequences and 30169 of the genes from this list was recognized. 14,952 duplicate genes were removed (keeping the highest ranked instance of each gene) leaving a total of 15,217 genes. The GOrilla output was saved to a flat file with Excel, then processed with command line tools/a trivial regex Python script. he data was visualized with GOPlot (1.0.2)^51^ and R (version 3.3.2). Results were compared topGO^52^ and qualitatively similar.

### RNA extraction

RNA extractions on *w1118* and *dan*^*AC116*^ flies were performed as described in Li and Barish *et al*.^13^.

### qRT-PCR

qRT-PCR from antennal RNA samples were performed for OR, GR, and IR genes in wild type and *dan* mutants. The primers used for OR and IR genes have been described in Li and Barish *et al*.^13^.

## Additional Information

**How to cite this article**: Barish, S. *et al*. Transcriptional profiling of olfactory system development identifies *distal antenna* as a regulator of subset of neuronal fates. *Sci. Rep.* **7**, 40873; doi: 10.1038/srep40873 (2017).

**Publisher's note:** Springer Nature remains neutral with regard to jurisdictional claims in published maps and institutional affiliations.

## Supplementary Material

Supplemental Figures and Legend

Supplemental Tables
