# Supplementary material for: Transcriptional profiling of olfactory system development identifies distal antenna as a regulator of subset of neuronal fates
Source: Sci Rep. 2017 Jan 19;7:40873. doi: 10.1038/srep40873 (PMC5244397; doi:10.1038/srep40873)
Supplement: Supplemental Figures and Legend [file srep40873-s1.pdf]

Transcriptional profiling of olfactory system development identifies *distal antenna* as a regulator of subset of neuronal fates

Supplemental Figures and Legends

Scott Barish[1], Qingyun Li[1,ξ], Jia W. Pan[1], Charlie Soeder[2], Corbin Jones[2,3], and Pelin C. Volkan [1,4]

[1] Duke University, Department of Biology, Durham NC

[2] University of North Carolina- Chapel Hill, Integrative Program for Biological & Genome Sciences, Chapel Hill, NC

[3] University of North Carolina- Chapel Hill, Department of Biology, Chapel Hill, NC

[4] Duke Institute for Brain Sciences, Durham, NC

[ξ] Current Address: Stanford University, Department of Neurobiology, Palo Alto, CA

Corresponding Author

Email: pc72@duke.edu (PCV)

Table S1. Transcripts with the top 50 scores for PC1 from PCA. Red line demarcates those transcripts with an absolute value greater 0.1 and those reported in the text.

Table S2. Transcripts with the top 50 scores for PC2 from PCA. Red line demarcates those transcripts with an absolute value greater 0.1 and those reported in the text.

Table S3. Significantly differentially expressed genes between 3L and p8 stages. Table of significantly differentially expressed genes ($p>10^{-6}$) between 3L and p8, represented by red dots in Fig 1B.

Table S4. Significantly differentially expressed genes between p8 and p40 stages. Table of significantly differentially expressed genes ($p>10^{-6}$) between p8 and p40, represented by red dots in Fig 1C.

Table S5. Significantly differentially expressed genes between p40 and Adult stages. Table of significantly differentially expressed genes ($p>10^{-6}$) between p40 and Adult, represented by red dots in Fig 1D.

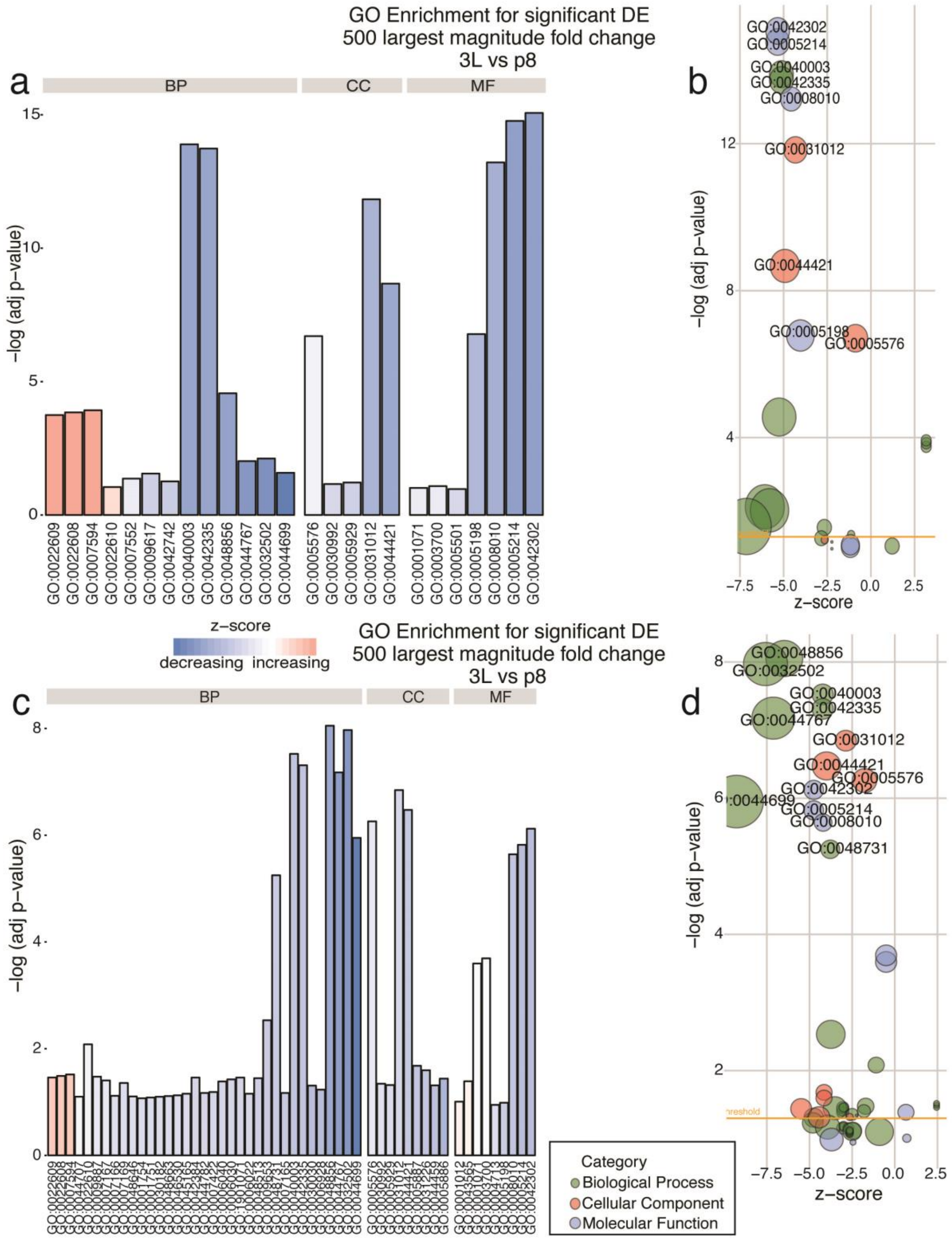

S1 Fig. Pairwise GO plot analysis of differentially expressed genes between 3L and p8 stages. Bar graphs and bubble plots showing enrichment of GO terms for the 500 genes with the largest fold change (A, B) and the 500 genes with the smallest p-values (C, D). Key for GO term numbers is located in Table S4. Green = Biological Process, Red = Cellular Component, Blue = Molecular Function.

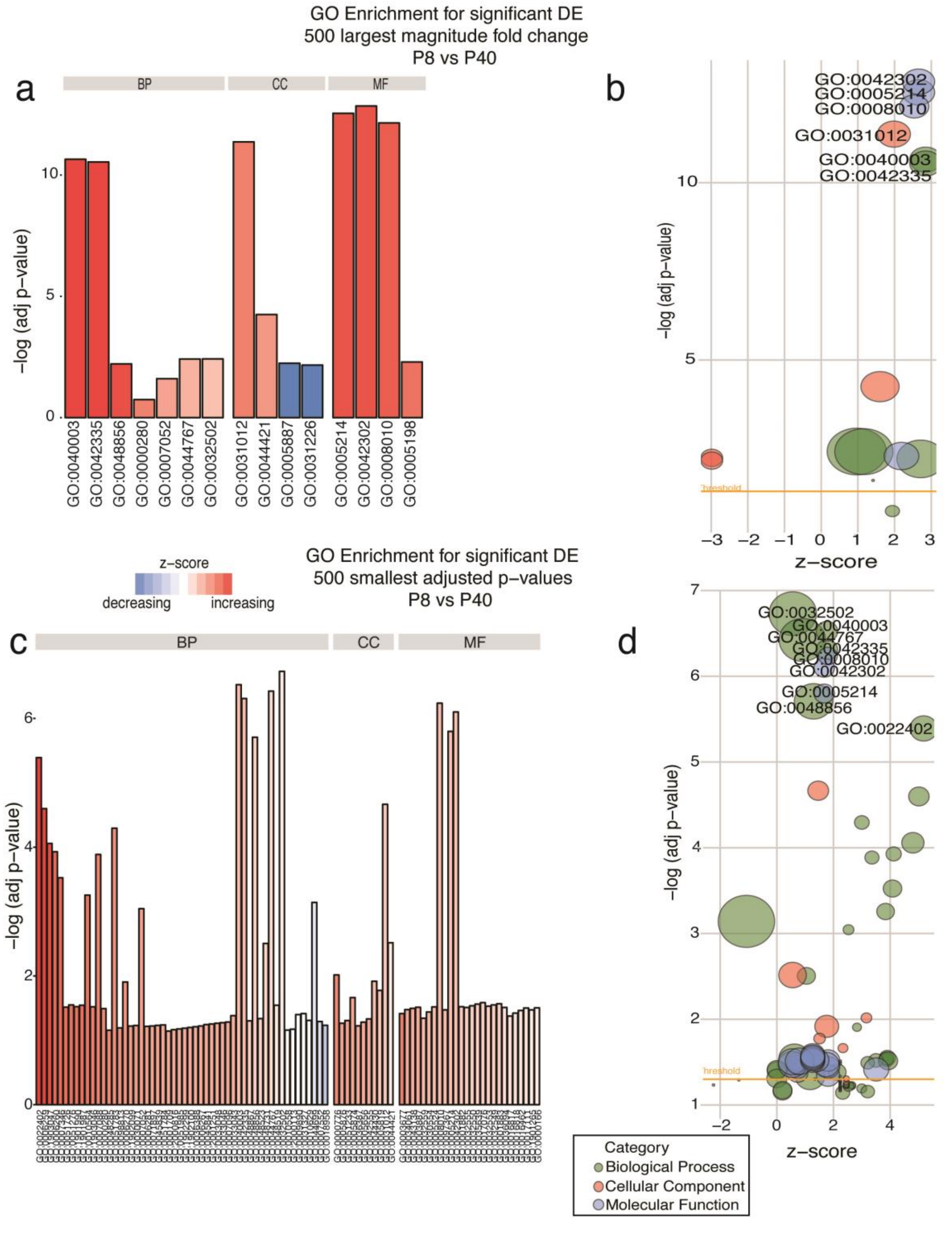

S2 Fig. Pairwise GO plot analysis of differentially expressed genes between p8 and p40 stages. Bar graphs and bubble plots showing enrichment of GO terms for the 500 genes with the largest fold change (A, B) and the 500 genes with the smallest p-values (C, D). Key for GO term numbers is located in Table S5. Green = Biological Process, Red = Cellular Component, Blue = Molecular Function.

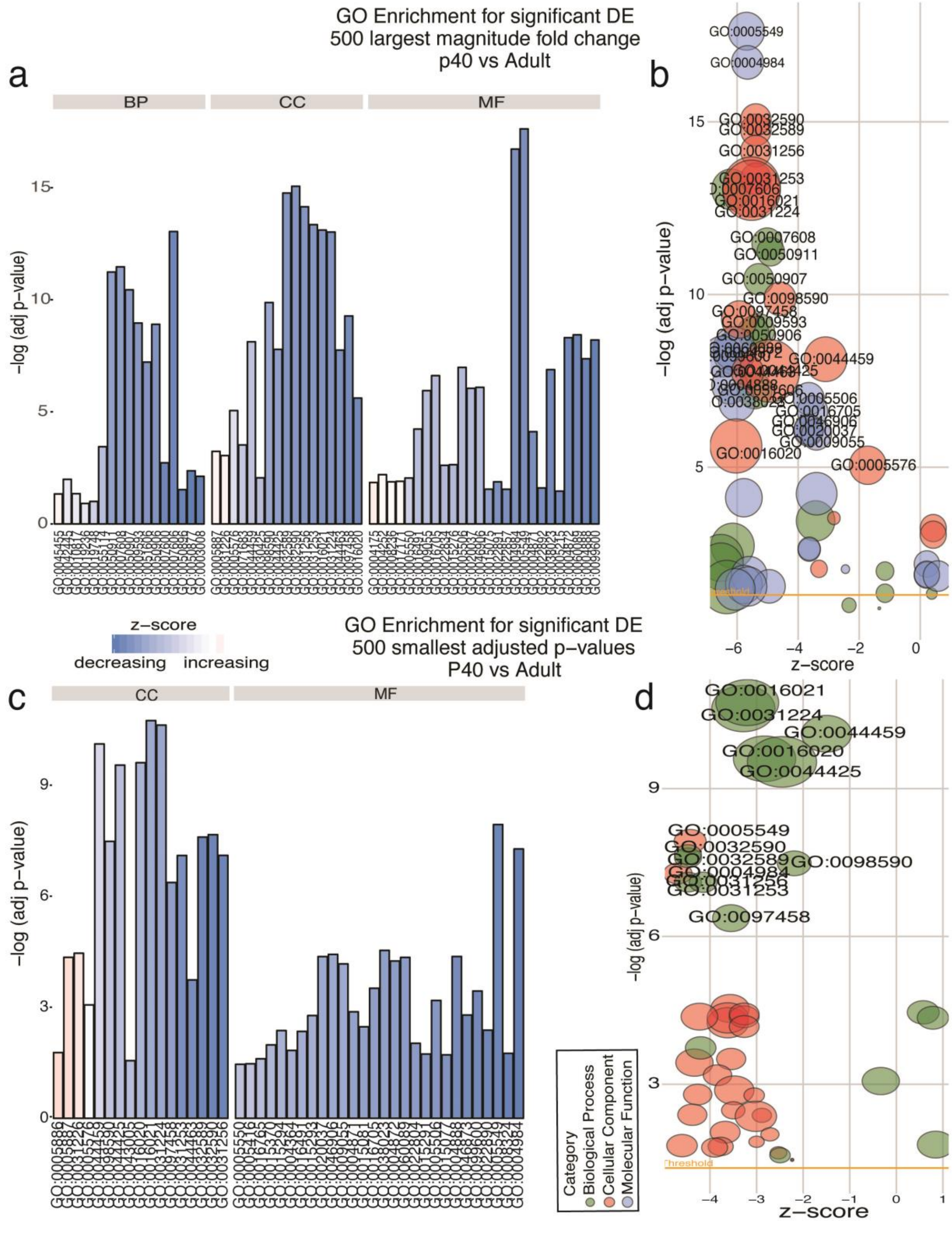

S3 Fig. Pairwise GO plot analysis of differentially expressed genes between p40 and Adult stages. Bar graphs and bubble plots showing enrichment of GO terms for the 500 genes with the largest fold change (A, B) and the 500 genes with the smallest p-values (C, D). Key for GO term numbers is located in Table S6. Green = Biological Process, Red = Cellular Component, Blue = Molecular Function.

Table S6. Key GO Terms in S1 Fig. Translation of GO numbers into terms for S1 Fig.

Table S7. Key GO Terms in S2 Fig. Translation of GO numbers into terms for S2 Fig.

Table S8. Key GO Terms in S3 Fig. Translation of GO numbers into terms for S3 Fig.

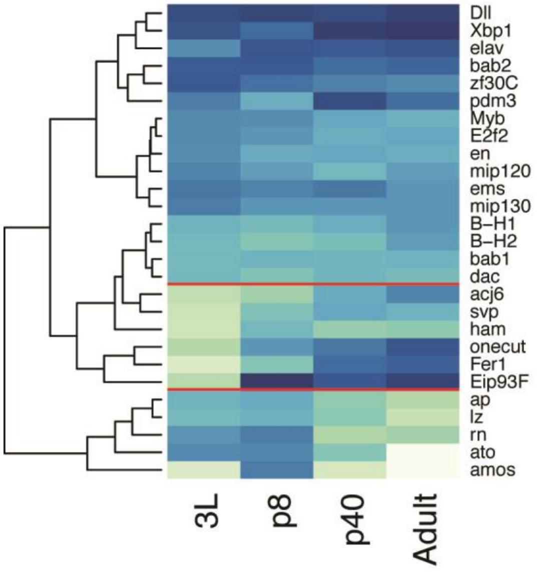

S4 Fig. Hierarchical clustering of the expression patterns of transcription factors known to regulate ORN specification. Transcription factors in the olfactory system are expressed generally in accordance to their function. Prepatterning and pronueral factors cluster at the bottom of the heatmap, indicating high expression early in development and low expression later. Terminal selectors on the other hand cluster just above, corresponding to low expression early and high expression late in development. Genes that cluster at the top of the heat map are expressed highly throughout development and fall into both categories.

Table S9. Normalized log_2 expression values of olfactory receptors used to create Figure 2. Values for the expression of all antennal ORs are listed including the olfactory co-receptor *orco*.

Table S10. Notch State and Expression Status of ORs Across Development. Table displaying the Notch state (adapted from Endo et al., 2007) and expression status for each OR at all developmental stages.

Table S11. Normalized log_2 expression values of transcription factors used to create Figure 4. Values for the expression of all transcription factors extracted from Flybase are listed.

Table S12. List of genes present in each cluster of Figure 4.

Table S13. Normalized log_2 expression values of commonly used housekeeping genes.
